# Associations of Body Composition and Mitochondrial Function in Older Adults

**DOI:** 10.1093/geroni/igab046.485

**Published:** 2021-12-17

**Authors:** Peggy Cawthon, Paul Coen, Bret Goodpaster, Russell Hepple, Stephen Kritchevsky, Anne Newman, Steve Cummings

**Affiliations:** 1 California Pacific Medical Center, San Francisco, California, United States; 2 AdventHealth, Orlando, Florida, United States; 3 University of Florida, Gainsville, Florida, United States; 4 Wake Forest School of Medicine, Winston Salem, North Carolina, United States; 5 University of Pittsburgh, Pittsburgh, Pennsylvania, United States

## Abstract

SOMMA assessed body composition by whole body magnetic resonance imaging (MRI) scans analyzed by AMRA (fat-to-weight ratio, mean: 57% ± 0.1; thigh muscle volume, mean: 8.9L ± 2.4; weight-to-muscle volume ratio, mean: 9.0 kg/L ± 1.6). Deuterated creatine dilution (D3Cr muscle mass, mean: 21.8kg ± 6.8) was used to measure muscle mass. Mitochondrial function was measured in fresh muscle tissue by respirometry (maximum OXPHOS and maximum ETS) and non-invasively by 31PMRS (ATPmax). Maximum OXPHOS and maximum ETS capacity were positively correlated with thigh muscle volume (r=0.42 and 0.44, respectively; p<.001) and D3Cr muscle mass (r=0.37 and 0.33, respectively; p<.001) and negatively correlated with fat-to-weight ratio (r=-0.36 and 0.26, respectively; p<.001) and weight-to-muscle volume ratio (r=-0.42 and -0.37, respectively; p<.001). Stratified analyses suggest that some associations may differ by sex. Associations between ATPmax and these body composition measures were more modest.

